# Methods used to design audit and feedback interventions to change clinical practice: a scoping review

**DOI:** 10.3389/frhs.2026.1907196

**Published:** 2026-09-15

**Authors:** Raquel Gallardo-Aviles, Mª Angeles García-Lirola, M. C. Gonzalez-Lopez, Regina Sandra Benavente Cantalejo, Carlos Barreda Velázquez, Rebeca Isabel-Gómez, Juan Francisco Gutierrez-Gil, M. Concepción Pérez Guerrero, Teresa Molina-Lopez

**Affiliations:** 1 Departamento de Farmacología, Universidad de Sevilla, Seville, Spain; 2 Servicio de Farmacia, Distrito Sanitario Huelva-Costa, Servicio Andaluz de Salud, Junta de Andalucía, Huelva, Spain; 3 Servicio de Farmacia, Distrito Granada, Servicio Andaluz de Salud, Junta de Andalucía, Granada, Spain; 4 Servicio de Farmacia, Distrito Almería, Servicio Andaluz de Salud, Junta de Andalucía, Almeria, Spain; 5 Subdirección de Farmacia y Prestaciones, Servicio Andaluz de Salud, Junta de Andalucía, Sevilla, Spain; 6 Dirección Asistencial Norte de Madrid, Servicio Madrileño de Salud, Madrid, Spain; 7 Área de Evaluación de Tecnologías Sanitarias, Fundación Pública Andaluza Progreso y Salud, Consejería de Salud y Consumo, Sevilla, Spain; 8 Servicio de Farmacia, Distrito Sevilla, Servicio Andaluz de Salud, Junta de Andalucía, Sevilla, Spain

**Keywords:** audit and feedback, health care, implementation science, intervention design, knowledge translation, theoretical framework

## Abstract

**Background:**

To optimize interventions that modify clinical practice, developers should use a systematic design process based on established frameworks. Reviewing current design practices in published literature and understanding established methodological guidelines is essential to identify gaps in knowledge, areas for improvement and promote best practices among interested groups.

**Objectives:**

This scoping review aims to synthesise the evidence on systematic design processes and methodologies for designing healthcare interventions published in articles and grey literature in the last fifteen years.

**Methods:**

Following PRISMA guidelines, we searched MEDLINE, Embase, and PsycINFO databases for peer-reviewed studies (April 2015–October 2025) describing in detail the selected framework and design process used. We also searched selected websites for published grey literature (from 2011 to October 2025) detailing specific intervention frameworks and design processes. Documents were excluded if healthcare professionals were not among the target recipients, intervention stages were poorly described, or the focus was exclusively on lifestyle habit changes. A narrative synthesis of findings was conducted.

**Results:**

We analysed 59 source documents (49 scientific studies; 10 grey literature documents). Across the studies, we identified up to 22 distinct theoretical frameworks (used alone or in combination) and between 2 and 10 process stages. In grey literature documents we identified five different theoretical frameworks and outlined between 4 and 11 stages. The inclusion of sustainability and implementation planning represents a new stage compared to models from 15 years ago, with piloting also emerging as a new, albeit less frequently reported, stage.

**Conclusion:**

Variability was identified in the theoretical models used to guide intervention design, which may be applied individually or in combination. Up to eleven stages were identified in the intervention design process. Greater methodological rigour and transparency in study reporting would be needed to improve replicability. Future research areas identified include assessing the impact of systematic design on effectiveness and cost-effectiveness, prioritising the development of de-implementation guidelines, and incorporating diverse, multidisciplinary perspectives into intervention design guidance documents, among others.

**Systematic Review Registration:**

https://doi.org/10.17605/OSF.IO/T5ZSX.

## 1 Introduction

The implementation of quality improvement interventions designed to optimise health outcomes and performance in healthcare organisations is a complex, resource-intensive process requiring multi-stakeholder engagement at various levels (1–3). Over the past decades, a substantial body of evidence has been generated on the effectiveness of interventions aimed at promoting changes in clinical practice. Initiatives such as the Cochrane Effective Practice and Organisation of Care (EPOC) Group have played a key role in synthesising and disseminating this evidence, supporting both the design of new interventions and the identification of research gaps (4).

Audit and Feedback (A&F) is one of the most widely utilised approaches for promoting change in clinical practice with some evidence of effectiveness. While numerous studies have introduced innovative designs to enhance the effectiveness of A&F-based interventions (5–8), overall improvements remain modest, leaving significant uncertainty on how to consistently maximise results. In terms of the absolute impact on clinical practice, recent estimates suggest that A&F typically improves compliance with desired behaviours by a modest 2.7% (interquartile range: 0.0%–8.6%) and that maximum effectiveness is achieved when implemented as part of a multi-component intervention (9).

In 2016 Brehaut et al. (7) proposed that following a systematic design process based on theoretical behavioural change theories could enhance the effectiveness of A&F interventions. A systematic review of fifteen intervention design studies published between 2001 and 2014 found that despite high variability in the processes described, four key elements were consistently present: identifying barriers, tailored component selection, applying a theoretical framework, and involving the target recipient in the design process (10). The authors underscored the need for further research into intervention design processes to establish methodological guidelines and identified and prioritised areas of A&F that should be the focus of research (11, 12). Many of these have been studied in the last decade, with the results recently reviewed and meta-analysed. The meta-analysis highlighted three factors for enhancing A&F effectiveness. First, A&F works best when it incorporates clear, specific action plans (9), particularly regarding process indicators (13), though this impact does not appear to extend to health outcomes (14). Second, effectiveness increases when feedback includes tailored benchmarking against effective comparators (9). This involves providing personalised comparisons (15) (rather than comparing everyone against a mean), tracking performance trends, setting targets, and encouraging feedback recipients to set personal, explicit goals guided by relevant information. Finally, the impact is greater when patient experiences are incorporated and the cognitive load required to interpret feedback is minimised (16). Research is currently being conducted into the effectiveness of precision A&F. This approach provides personalised feedback and dynamically adjusts the feedback mechanism based on previous cycle results (17).

In parallel, a wide range of theoretical frameworks, models, practical guidelines have been developed to support the design and implementation of complex interventions (1, 18–25) and proposals have been made to establish partnerships and collaborations between healthcare organisations interested in systematically designing A&F-based interventions on a larger scale (26).

In this scenario some knowledge gaps remain. First, it is unclear how intervention design methods have evolved over time. Although earlier work identified key components of design processes, there has been no comprehensive synthesis of how these methods have developed in the past fifteen years, particularly following the last systematic review in this area (8). Understanding this evolution is essential to determine whether advances in theory and methodology have translated into changes in practice.

Second, there is no integrated overview of what guideline documents exist overall, which theoretical approaches are most commonly promoted, into what stages the design process is structured, and how consistent these recommendations are across sources.

To address these gaps, this scoping review aims to: (1) map the scientific literature on the design of A&F interventions and describe how design approaches have evolved over the past fifteen years since the last systematic review on this topic; and (2) identify and synthesise existing design guidelines, frameworks, and practical manuals for developing A&F and related implementation interventions through a review of grey literature.

## 2 Methods

This study was originally designed and registered as a systematic review (OSF https://doi.org/10.17605/OSF.IO/T5ZSX). However, during the early stages of study selection and data charting it became clear that the available evidence was heterogeneous in terms of study design, objectives, and reporting of intervention design processes with several studies not reporting measures of effectiveness. This circumstance, together with the fact that effectiveness outcomes were not central to our research questions, further supported our decision not to conduct a formal quality appraisal. In addition, a substantial component of this review involves grey literature, including guidance documents and design manuals. For these types of sources, there were no widely accepted or standardised tools for assessing methodological quality comparable to those used for empirical studies, which further limits the appropriateness of conducting a formal quality appraisal across all included evidence. In these circumstances, the protocol was amended to reflect a scoping review approach, and for the reporting we followed the PRISMA-ScR guidelines (27) (Supplementary File 1: PRISMA-ScR checklist).

### 2.1 Review questions

For the scoping review of scientific literature:

1. What theoretical frameworks or models have been used to guide intervention design processes aimed at modifying clinical practice habits in recent years?
2. What specific stages, tasks or iterative processes are described in the recent scientific literature for designing interventions to modify clinical practice habits?

For the scoping review of grey literature:

1. What manuals or guidelines for intervention design have been published in recent years?
2. What theoretical frameworks do they use to guide this process?
3. In terms of the stages of the design process, what are the main characteristics and proposals described in these guidelines?

### 2.2 Search strategy

For the scientific literature review, an information professional conducted a comprehensive literature search of the MEDLINE, EMBASE and PsycINFO databases for English or Spanish studies published between April 2015 [the search end date of the previous review (8) and October 2025 focusing on interventions designed to modify the behaviour of healthcare professionals (Supplementary File 2 details the full search strategies used in each database and search cycles).

For the grey literature, three sources of information were explored to identify documents or guidelines for intervention design published between January 2011 and October 2025. These sources were: a) websites of agencies or organisations focused on healthcare quality improvement, implementation and health technology assessment; b) websites of implementation research projects; c) the first ten pages of Google search results, using the search terms: “Implementation Guidelines”, “Audit and Feedback Design”, “Medical Audit Guidelines”, “Quality Improvement Guidelines”. The search was supplemented by a manual review of the reference lists of studies included in the scientific literature review. The list of screened websites can be provided upon request.

### 2.3 Inclusion and exclusion criteria

#### 2.3.1 Scientific literature

Studies were eligible for inclusion in the scientific literature review if they met the following criteria:

- Target population: The target recipients of an intervention included health professionals in any healthcare setting (primary care, hospitals, nursing homes);
- Study content and design: Studies described (in full or in part) the framework and process followed to design an intervention intended to improve or sustain professional healthcare practice. The intervention included A&F as a component. All study designs, comparators and outcomes were eligible. Protocols and systematic reviews were included provided that they reported on the intervention design.

Studies were excluded if they met any of the following criteria: Healthcare professionals were not included among the intervention recipients (i.e., the intervention was targeted exclusively at patients); the intervention framework or stages were not clearly described; the focus was exclusively on changes in lifestyle habits (diet, exercise, smoking cessation, alcohol and drug abuse cessation).

#### 2.3.2 Grey literature

Documents were eligible for inclusion in the grey literature review if:

- they detailed the full process or one or more actionable stages of intervention design;
- they provided sufficient procedural detail to enable any professional with basic implementation science skills to design an intervention or one specific stage of the intervention.

Documents were excluded if they focused exclusively on designing interventions to promote healthy living behaviours (diet, exercise, smoking cessation, alcohol and drug abuse cessation).

### 2.4 Selection process for scientific literature and grey literature

Following the literature search, the identified studies were exported to a reference management tool. The selection process was conducted by a team of six reviewers working in pairs (TML, RGA, RSBC, CBV, MAGL, JFGG). The first ten articles were reviewed jointly by all six reviewers to ensure inter-rater procedural consistency and reliability. Next, pairs of reviewers screened titles and abstracts, followed by independent full-text assessment of all eligible studies to confirm the inclusion criteria. Disagreements were resolved by consensus within each pair. In cases of disagreement, a third reviewer was consulted until consensus was reached.

Following initial article selection, two members of the research group manually searched the reference lists of included published studies to identify further relevant articles and grey literature not previously found through database or website searches.

MCGL and TML independently screened titles, abstracts and executive summaries of grey literature against inclusion. Disagreements were resolved through discussion. When initial pairs could not reach a consensus, a third reviewer was consulted until agreement was reached. Full texts of potentially relevant guidelines were retrieved and reviewed against the inclusion criteria. To ensure inter-rater reliability, reviewers piloted the first three studies jointly.

### 2.5 Data extraction

Data extraction was conducted by team members working in pairs (TML, RGA, RSBC, CBV, MAGL, JFGG, for scientific articles; TML and MCGL for grey literature) using data extraction forms. To ensure accuracy, reviewers pilot-tested the forms on five studies and two guidelines. Discrepancies were resolved in pairs through discussion, consulting a third reviewer, when necessary, until agreement was reached.

Key data extracted from the scientific literature included: first author, year of publication, funding source, country, healthcare setting, study design, purpose/target behaviour of the intervention (28). We also documented the theoretical frameworks used, the stages of the intervention process described, together with their main details, and whether the authors used established forms or guidelines for the structured reporting of intervention design. These included: WIDER (Workgroup for Intervention Development and Evaluation Research) (29), TIDiER (30), Proctor (31, 32), StaRI [Standards for Reporting Implementation Studies (33)] or GUIDED (Guidance for Reporting Intervention Development Studies in Health Research) (34).

Source details extracted from the grey literature included: publication date, funding source, country, healthcare organisation responsible for development. Content details included: theoretical frameworks employed in the design process, scope (entire design process versus specific stages), and the main characteristics of each stage.

After the initial data extraction, we conducted an updated search (up to October 2025) to capture relevant new scientific studies and grey literature published while drafting this manuscript.

Data extracted from the included studies and grey literature are available upon request.

### 2.6 Critical appraisal of individual sources of evidence

Critical appraisal of the included scientific studies and grey literature documents — including risk of bias and methodological quality — was not undertaken. This decision is consistent with established scoping review methodology, in which critical appraisal is considered an optional component (27), given that the aim of a scoping review is to map the breadth and nature of the available evidence rather than to synthesise its strength or determine the certainty of effect estimates.

### 2.7 Data synthesis and analysis

A narrative synthesis of the selected studies and grey literature was conducted, supported by a table detailing key source characteristics. No further quantitative synthesis was performed.

## 3 Results

Of the 2,104 scientific articles identified, 313 were selected for full text review, with 49 studies ultimately meeting the criteria for inclusion (35–83). The excluded studies, together with the reasons for their exclusion, could be provided under request. The grey literature search yielded 91 documents. Following an initial screening based on title, year of publication and indices, 65 were excluded. Twenty-six documents were selected for full-text review, of which 16 were excluded for various reasons. The reasons for exclusion are available upon request. This process left 10 documents selected for analysis (3, 21, 25, 84–90). The process for identifying and selecting scientific articles and grey literature is shown in Figure 1 (91).

**Figure 1:**
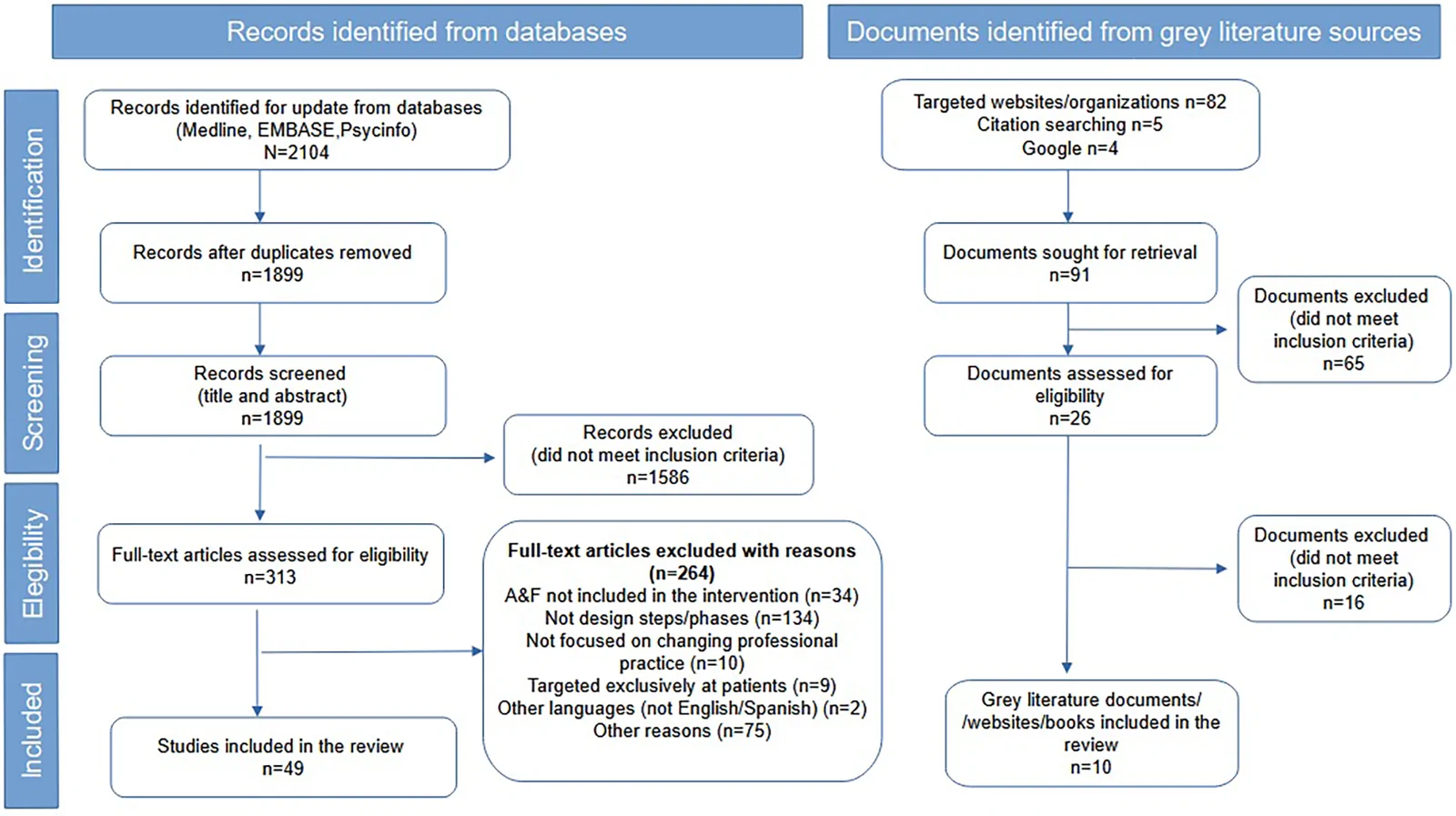
PRISMA flow diagram. Adapted from Page et al. (91). Licence CC BY 4.0.

### 3.1 Characteristics

Table 1 describes geographical origin and health settings for scientific articles. Figure 2 shows the number of articles by year of publication.

**Figure 2:**
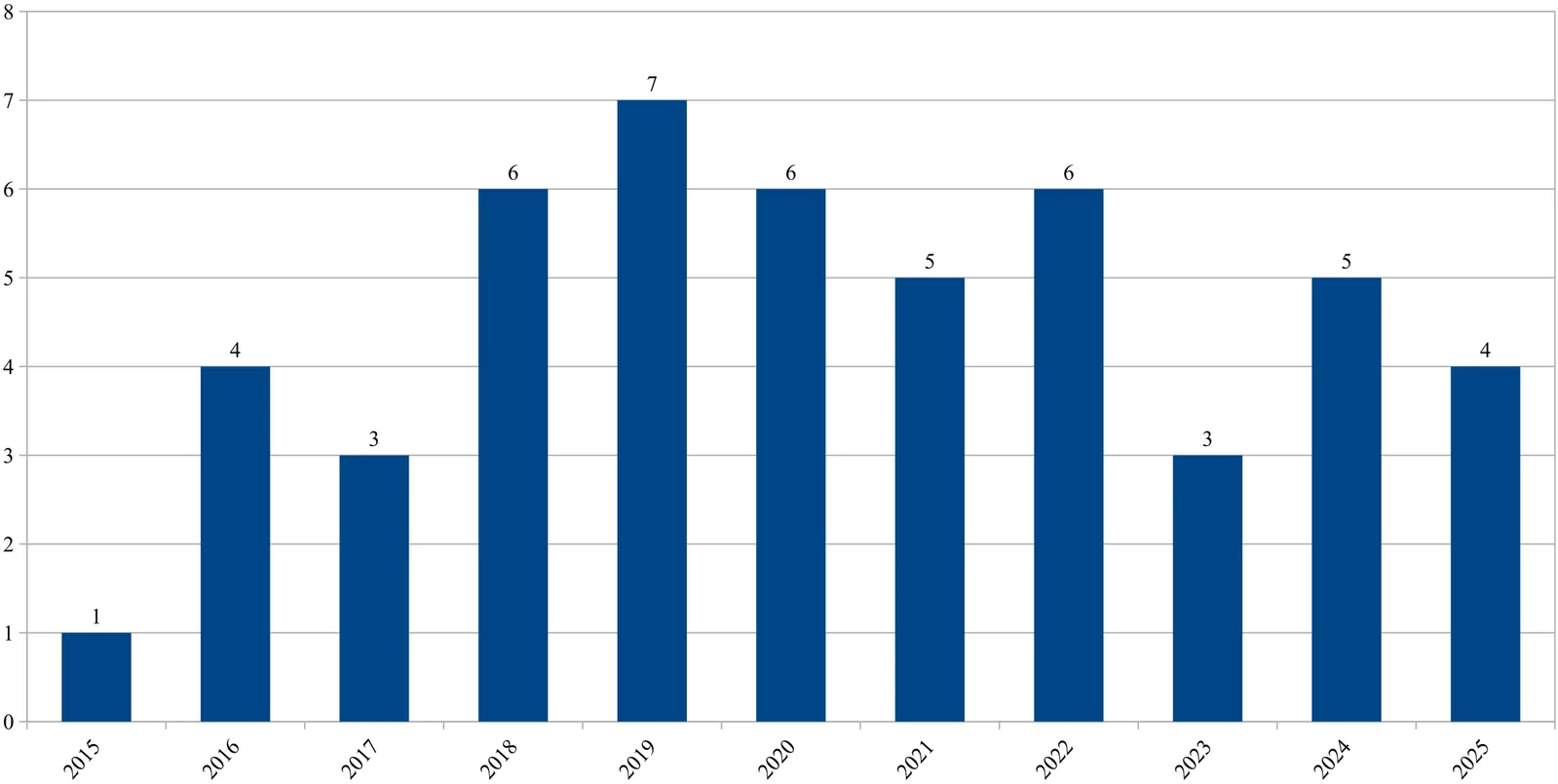
Number of included studies by year of publication.

**Table 1:** Geographic origin and healthcare settings of included studies.

Country	% (n/N)	References
Australia	20% (10/49)	(38–40, 46, 49, 57, 61, 63, 65, 81)
USA	17% (8/49)	(36, 44, 45, 48, 50, 55, 70, 77)
China	10% (5/49)	(51, 52, 72, 79, 83)
Canada	8% (4/49)	(37, 58, 80, 82)
Spain	8% (4/49)	(43, 53, 54, 71)
UK	6% (3/49)	(73, 74, 78)
Brazil	4% (2/49)	(47, 56)
Ireland	4% (2/49)	(41, 60)
Netherlands	4% (2/49)	(42, 59)
Portugal	4% (2/49)	(68, 69)
Argentina	2% (1/49)	(62)
Australia-New Zealand	2% (1/49)	(64)
Belgium	2% (1/49)	(75)
Kenya-Nigeria-South Africa-Tanzania	2% (1/49)	(76)
Saudi Arabia	2% (1/49)	(66)
South Africa	2% (1/49)	(67)
Uganda	2% (1/49)	(35)

Healthcare settings	% (n/N)	References
Hospital^a^	71% (35/49)	(35–40, 42, 43, 46–48, 50–52, 54–56, 59, 61–70, 72, 73, 76, 77, 80, 81, 83)
Primary care	27% (13/49)	(41, 44, 45, 49, 53, 57, 58, 60, 71, 74, 78, 79, 82)
Nursing home	2% (1/49)	(75)

USA: United States of America; UK: United Kingdom.

a includes one article conducted in a geriatric outpatient department (59) and one article conducted in a midwife obstetric unit (67).

Regarding the study designs, quantitative designs, such as experimental or observational before-and-after studies, were used to measure the effectiveness of the intervention. Mixed-methods approaches were employed to address multiple research objectives. Qualitative methods (e.g., focus groups and semi-structured interviews) and quantitative techniques (e.g., surveys) were used to establish the baseline situation, identified barriers and facilitators and selected intervention components.

The target behaviours addressed in the included studies are shown in Table 2, and are categorised based on the Cochrane Effective Practice and Organisation of Care (EPOC) (28) Most studies (80%; 39/49) reported public funding. Only four studies (8%; 4/49) declared no external funding or grant support (56, 65, 73, 77), whereas the funding status was not reported in six studies (12%; 6/49) (36, 40, 42, 47, 48, 54).

**Table 2:** Target behaviours identified in included studies and number addressed per intervention.

Target behaviours^a^	% (n/N)	References
General management of a problem	61% (30/49)	(36, 37, 40–44, 48, 50, 52, 54, 57, 58, 61–64, 67, 69, 71–74, 76–80, 82, 83)
Prescribing	31% (15/49)	(41, 42, 44, 49, 50, 57, 58, 62–64, 66, 71, 75, 76, 79)
Patient education/advice	29% (14/49)	(38, 40, 43, 45, 47, 52, 57, 64, 74, 77–79, 82, 83)
Clinical prevention services	20% (10/49)	(36, 38, 43, 45, 47, 49, 65–68)
Diagnosis	16% (8/49)	(36, 39, 51, 57, 67, 72, 76, 79)
Discharge planning	2% (1/49)	(83)
Professional-patient communication	2% (1/49)	(59)
Referrals	2% (1/49)	(46)
Others^b^	8% (4/49)	(35, 53, 55, 60)
No. of target behaviours	% (n/N)	References
One	43% (21/49)	(35, 37, 39, 46, 48, 51, 53–56, 59–61, 65, 68–70, 75, 80, 81)
Two	41% (20/49)	(38, 40–42, 44, 45, 47, 49, 50, 52, 58, 62, 63, 66, 71, 72, 74, 77, 78, 82)
Three	12% (6/49)	(36, 43, 64, 67, 76, 83)
Four	4% (2/49)	(57, 79)

a Based on Cochrane Effective Practice and Organisation of Care (EPOC) Group. Cochrane Effective Practice and Organisation of Care Review Group. Data collection checklist (28).

b hand higiene, patient safety in primary care, reduction of length of stay in the emergency department, screening.

Table 3 describes the general characteristics of the grey literature documents.

**Table 3:** Grey literature documents. General description.

Title (Acronym)	Institution/ Organisation	Year	Country	Funding
Moving into action: We know what practices we want to change, now what? An implementation guide for health care practitioners (CIHR-MIA) (84)	Canadian Institutes of Health Research	2012	Canada	Canadian Institutes of Health Research
Michie et al. The Behaviour Change Wheel: A Guide to Designing Interventions (BCW-Guide) (21)^a^	Michie et al.	2014	United Kingdom	Silverback publishing (ed)
Greenhalgh T. How to Implement Evidence-Based Healthcare (IEBHC) (3)^a^	Greenhalgh T.	2018	United Kingdom	John Willey and Sons Ltd (ed)
Guidance on developing interventions to improve health and health care (SSB-Univ) (86)	Universities of Sheffield/Stirling/Bristol	2019	United Kingdom	Medical Research Council
JBI Handbook for Evidence Implementation (JBI) (25)	Joanna Briggs Institute	2020	Australia	University of Adelaide, Australia
Six Building Blocks: A Team-Based Approach to Improving Opioid Management in Primary Care: How-To-Implement Toolkit (6BB) (87)^b^	AHRQ	2021	USA	U.S. Department of Health and Human Services
Guia per a la implementació de projectes d'atenció sanitària basada en el valor (AQuAS) (88)	AQuAS	2021	Spain	Departament de Salut de la Generalitat de Catalunya
A brief guide to effective audit and feedback (A&F Leeds-Univ) (89)	University Leeds	2022	United Kingdom	NIHR
Evidence-based practice in action: Comprehensive strategies, Tolls and Tips from University of Iowa Hospitals and Clinics (EBPA-Iowa) (90)^a^	University of Iowa Hospitals & Clinics	2022	USA	Sigma Theta Tau International (ed)
Toolkit: implementation of best practice guidelines. (Second edition) (RNAO) (85)	Registered Nurses' Association of Ontario	2024	Canada	Ontario. Ministry of Health and Long-Term Care

Institution/ Organisation refers to who promotes the development of each document; NIHR: National Institute for Health Research; AHRQ: Agency for Healthcare Research and Quality; AQuAS: Agència de Qualitat i Avaluació Sanitàries de Catalunya.

a Documents not freely available.

b Project 6BB (Six Building Blocks) aims to promote the implementation of an opioid detoxification intervention. The intervention design is described in the published articles on the topic, which are listed in https://familymedicine.uw.edu/improvingopioidcare/publications-and-news/.

### 3.2 Theoretical frameworks

Table 4 summarises the theoretical frameworks and design processes described in the articles included in the review. Although a wide variety of frameworks was identified, three approaches predominated: the Behaviour Change Wheel (BCW), used independently or in combination, in 37% (18/49); the Theoretical Domains Framework (TDF), alone or in combination, in 33% (16/49) and Getting Research into Practice (GriP), in 24% (12/49) of studies.

**Table 4:** Summary of intervention design process in selected scientific literature.

Frameworks	No. stages by authors	Team (T/L)	Basal situation	Problem definition (P/TB)	Evidence	Barriers-Facilitators	Components (C/R)	Feasibility (F)/ Aceptability (A)	Pilot	Evaluation (E) Monitoring (M)	Sustainability
BCW (49)	5			P/TB		●	C	F/A	●		
BCW-BCD (70)	NE	TL				●	C		●		●
BCW-MRC (57)	3	T		P/TB	●	●	C				
BCW-DSRP-MRC (41)	3			TB	●	●	C				
BCW-KTA (65)	NE		●			●	C, R			E	
BCW -IPARIHS-MRC (74)	4	T			●	●	C, R		●	E	●
BCW-TDF (46)	6	T	●	TB		●	C			M	
BCW-TDF (58)	3	T	●			●	C			E	
BCW-TDF (64)	3			TB	●	●	C, R				
BCW-TDF (71)	8	T		P/TB		●	C				
BCW-TDF (75)	4	T		TB		●	C, R	F	●	E	
BCW-TDF (81)	3					●	C		●		
BCW-TDF (82)	2					●	C, R				
BCW-TDF (83)	4			TB		●	C, R			E^a^	●
BCW-TDF-MRC (76)	2				●	●	C, R	F/A	●	E	
BCW-TDF-MRC (78)	3	T					C	F	●		
BCW-TDF-CFIR (45)	3	T				●	C, R	A^b^			
BCW-TDF-CFIR (60)	4		●	TB		●	C	F			
TDF (39)	6	T	●	TB		●	C			E	
TDF (48)	5		●			●	C			E	
KTA-CFIR-TDF (73)	2					●	C, R				
CICI-TDF-Proctor (67)	3		●			●	C	F/A	●	E	●
GriP (35)	3	TL	●		●	●	C, R			M	
GriP (38)	NE	TL	●			●	C, R			M	●
GRiP (40)	3	TL	●			●	C			M	●
GriP (43, 47, 51, 52, 56)	3	TL	●			●	C, R			M	
GriP (54)	3	TL	●			●	C, R			M	●
GriP (61)	3	TL	●			●	C, R	A^b^		M	
GriP (68, 69)	3	T	●			●	C, R			M	
MRC-FIT (53)	3					●	C	F/A	●	E	
MRC (59)	3	T				●	C	F	●		
MRC-CFIR (80)	NE					●	C	A^c^			
KTA (37)	7	TL	●	P/TB	●	●	C	F	●	M/E	●
KTA (72)	4				●	●	C			M/E	
CFIR (50)	NE	TL	●		●	●	C, R			E	●
DMAIC (36)	NE	TL	●	P/TB	●		C			M/E	●
Cabanás CPGFI + Nielseńs Heuristics (44)	NE					●	C	A	●		
HCD (55)	NE					●	C		●	E	
iPARIHS (62)	3	TL	●			●	C, R			E	
Quality improv. (66)	3	TL			●		C			E	
Quality improv (63)	3	L	●			●	C			E	
Iowa ISF-Impl Strat EBPG (77)	NE	TL			●		C		●	E	●
Control Theory (42)	NE		●			●	C	A^c^			
WHO 6BB-WHO people centered (79)	3	T		P/TB		●	C				

The following terms and abbreviations were used for each stage, with a brief description of each:.

No. of stages identified by authors in the included articles; Team (T/L): T denotes formation of a multidisciplinary team for intervention design; L denotes leadership; Baseline situation: the article included an assessment of the baseline situation of the practice needed to be modified; Problem definition (P/TB): *P* indicates a specific stage for defining the problem; TB indicates the identification and analysis of the target behaviour to be modified; Evidence: the article included an analysis or consideration of the evidence during the intervention design process; Barriers-Facilitators: recorded when the article analysed barriers and/or facilitators to changing clinical practice; Components (C/R): C was recorded when the article described the components of the intervention; R was recorded when the resources required to implement these components were specified; Feasibility/acceptability (F/A): recorded when the article assessed the feasibility (F) and/or acceptability (A) of the intervention; Pilot: recorded when the article included pilot testing of the intervention; Monitoring(M)/Evaluation (E):recorded when the article included plans for monitoring and/or evaluation of the intervention; Sustainability: recorded when the article addressed the sustainability of the intervention.

Frameworks identified: BCW, behaviour change wheel; HCD, human centred design; MRC, Medical Research Council design and evaluation of complex interventions framework; DSRP, design science research process; KTA, Knowledge to Action; iPARIHS, integrating Promoting Action on Research Implementation in Health Services; Quality improv, quality improvement; TDF, Theoretical Domains Framework; CFIR, Consolidated Framework Implementation Research; CICI, Context and Implementation of Complex Interventions; Proctor, Proctor's framework; GriP, Getting Research into Practice; FIT, Feedback Intervention Theory; DMAIC, Define-Measure-Analyse-Improve-Control; Cabana's CPGFI, Clinical Practice Guidelines Framework for Improvement; Nielsen's Heuristics, Nielsen's Usability Heuristics; Iowa ISF, Iowa implementation for sustainability framework; Impl Strat EBPG, Implementation Strategies for Evidence-Based Practice Guide; WHO-6BB, WHO six health system building blocks; WHO-people centered, WHO strategies for integrated people-centered health services.

a economic evaluation included.

b readability of the checklist for patients assessed.

c usability assessed.

Table 5 summarises the data extracted from the grey literature documents in relation to the intervention design process. Eight documents are structured as guides organised around the different stages of the intervention design process. A ninth document also takes the form of a guide, but is not structured into stages, as it focuses specifically on the design of an audit and feedback cycle. The tenth document is a book that provides a comprehensive and detailed review of the key elements involved in implementing innovations in clinical practice.

**Table 5:** Summary of intervention design process in selected grey literature documents.

Document	Framework	Team (T/L)	Basal situation	Problem definition (P/TB)	Evidence	Barriers-Facilitators	Components (C/R)	Feasibility (F)/ Acceptability (A)	Pilot	Evaluation (E) Monitoring(M)	Sustainability
Formatted as a stage-by-stage guide											
CIHR-MIA (84)	KTA	T L	●	P/TB	●	●	C R	F		E/M	●
BCW-Guide (21)	BCW		●	P/TB		●	C				
SSB-Univ (86)	Decided by the team	T	●	P/TB	●	●	C R	F/A	●	E	●
JBI (25)	GRiP	TL	●	P/TB	●	●	CR			M	●
6BB (87)	Six building blocks	TL	●	P/TB	●	●	CR		●	M	●
AQuAS (88)	Not specified	TL	●	P/TB		●	CR			E	
EBPA-Iowa (90)	Iowa ISF		●	P/TB		●	C R	F	●	E/M	●
RNAO (85)	KTA	T	●	P/TB	●	●	CR		●	E/M	●
Other formats (*)											
IEBHC (3)	Decided by the team	TL	●	P/TB	●	●	C R			E/M	●
A&F Leeds-Univ (89)	Not specified			P/TB			C				

See Table 3 for acronyms and Table 4 for clarification of the headings; *IEBHC format is a book that provides a comprehensive and detailed review of the key elements involved in implementing innovations in clinical practice. The A&F Leeds-Univ format is a guide that focuses only on the design of an A&F cycle.

### 3.3 Stages in the design process

The original studies and the grey literature used varying terminology and described a number of design stages, ranging from two to ten in the studies and between four and 11 in the grey literature documents. To facilitate the analysis, we consolidated a coherent ten-stage process, using standardised terminology, based on the core actions described. Tables 4, 5 outline this mapping across the various studies and documents, respectively. The key aspects of each stage are presented below:

- Formation of a multidisciplinary team of professionals to design the intervention is an initial phase identified in 61% (30/49) of studies. Although not always listed as a formal stage, some studies explicitly describe forming a group (35, 38–40, 43, 51, 52, 54, 56, 61, 68, 69), with 37% (18/49) highlighting the need for structured leadership throughout the design process. Among the included grey literature documents describing the entire process (3, 25, 84–88, 90), some devote specific sections to this aspect (3, 25, 84–88, 90), whereas others integrate the best practices recommendations throughout the text (21, 89). Best practices include assembling a multidisciplinary team, clearly defining leadership roles (25, 84, 85, 87, 88, 90), and actively involving all relevant stakeholders, including patients. These practices are defined as essential for fostering effective communication, maintaining positive group dynamics (3), and ensuring institutional support (3, 88).
- Assessment of the baseline situation and identifying and defining the clinical issue to be improved. The second stage involves evaluating the initial baseline conditions. This stage was reported in 51% (25/49) of the studies, primarily conducted through baseline audits, and was described in most of the grey literature documents (3, 21, 25, 84–88, 90).
- Problem and objective definition. This stage involves explicitly defining the target clinical behaviours and establishing clear objectives for the intervention. In scientific literature this is achieved by engaging stakeholders through semi-structured interviews and collaborative workshops (27%; 13/49). All grey literature documents recommend stakeholders collaboration in this stage and some of them include specific tools to assist with the definition of objectives (21, 84, 85, 87, 90).
- Gathering and synthesising evidence and recommendations that justify and support the intervention. This stage, focuses on evidence-based, pre-implementation preparation to ensure that the proposed intervention is scientifically sound and tailored to the local context. It includes activities such as identifying and reviewing clinical practice guidelines to help define the problem area or select target behaviours (36, 41, 77), as well as provide evidence to support the change in practice promoted by the intervention (35, 37, 50, 57, 64, 66, 72, 74, 76). Review of evidence also help in other stages of the design such as identifying local barriers and facilitators of change (37, 41, 50, 52, 57, 59, 63, 75, 79, 80), developing the necessary resources, materials or tools (42) and selecting core components of the intervention (14, 53, 70, 74, 78, 83). Grey literature documents that address this stage treat evidence gathering to support the intervention and adaptation to the local context as separate, sequential stages (84–87), although in the case of Knowledge-to-Action (KTA) framework the processes are interdependent, evidence is synthesised and refined within the knowledge funnel while being simultaneously adapted and tailored to the local context within the action cycle. Some of them emphasize the importance of reviewing evidence regarding other similar interventions and their components, as this also helps define the problem or assess potential barriers and facilitators (86, 87) or even decide which stage needs to be developed anew.
- Identification of barriers to and facilitators of the intervention: This stage was found in 92% (45/49) of the studies. The procedures used for identification varied, ranging from synthesising evidence from published data on barriers and facilitators (37, 41, 50, 52, 57–59, 63, 70, 75, 76, 79, 80) to conducting *de novo* analyses using qualitative research methodologies (75). Other approaches included brainstorming (35, 52, 72), discussion of the baseline audit results (35, 38, 40, 43, 47, 51, 54, 56, 61, 68, 69), interviews (44, 46, 49, 50, 53, 55, 58–60, 64, 74, 76, 82, 83), focus groups (39, 46, 53, 60, 65, 71, 74, 82), root cause analysis (72), quantitative methods (surveys of patients or professionals (62, 70) or mixed methods (45, 48, 67, 73, 76, 81, 83) and others (14). This is a core stage across most grey literature documents (3, 21, 25, 84–88, 90) and some of them provide detailed guidance on the procedures required (3, 21, 84, 85, 87, 90).
- Identification of intervention components and prioritisation. All the reviewed studies described this stage. Several included a prioritisation process using APEASE criteria (acceptability, practicability, effectiveness, affordability, cost-effectiveness, side-effects and equity) to select components tailored to the specific context (41, 57, 60, 75, 81, 83) or considering feasibility, acceptability (64, 71, 76) and effectiveness (71) or local relevance (64). Similarly, all the grey literature documents address this stage. Two of them stand out for their detailed description of the selection process and tools they provide (21, 25) using also APEASE criteria. Four dedicate a section to discussing the A&F as a component (3, 25, 84, 85). One of the documents is dedicated exclusively and in depth to the design of this component (89).
- Assessment of the acceptability and/or feasibility of the intervention, or of a specific component or material. 29% (14/49) of the studies report on the acceptability of the intervention and whether it is appropriate, convenient or satisfactory for patients and professionals or on its feasibility, or practicality. In some studies, acceptability was limited to patient-facing documents (readability) rather than the intervention as a whole) (45, 61). Others assessed usability of the dashboard designed (14, 80). Among grey literature documents, three focus on the importance of assessing feasibility (84, 86, 90) or acceptability (86).
- Pilot study. 29% (14/49) of the studies and four grey literature documents (85–87, 90) included a pilot stage to evaluate feasibility and refine study design before large-scale implementation.
- Evaluation and/or monitoring procedures. Procedures for evaluation and/or monitoring of the intervention were defined as a specific stage in 65% (32/49) of the studies. Methodological approaches varied, including before-and-after audits (35–40, 43, 46, 47, 51, 52, 54, 56, 61, 68, 69, 72); interrupted time-series (65); quantitative and qualitative analyses (58, 63, 67); pre-post intervention survey (55), quasi-experimental before-and-after studies (66, 75, 77); randomised controlled clinical trials (53, 76); hybrid type II studies (74) or stepped-wedge hybrid type II trials (50). In grey literature documents these distinct but closely linked stages include creating indicators, measurement procedures and data sources to track and assess clinical practice changes.
- Development of a sustainability plan. This final stage was reported in 22% (11/49) of the studies. Key actions to be developed at this stage include the identification of new barriers (37, 40, 70), implementing cascade training (67) and identifying professionals who will act as champions to ensure that the effects of the intervention endure permanently (54). Grey literature documents (3, 25, 84–87, 90) recommend this plan be initiated from de beginning of the design process, and, one of them suggests that it should be integrated with the monitoring plan (87).

Seven grey literature documents also describe a stage for *Implementation planning* (3, 84–88, 90). Some documents describe an active communication plan throughout the entire process to communicate the intervention objectives, milestones and results to healthcare professionals and patients (3, 25, 84, 85, 88, 89).

Finally, 16% (8/49) of the studies employed standardised forms or guidelines to report interventions. The reporting tools or guidelines used included: TIDiER (Template for Intervention Description and Replication) (7 studies) (49, 57, 60, 64, 67, 75, 80), GUIDED (Guidance for reporting intervention development studies in health research) (3 studies) (75, 78, 80); Proctor (2 studies) (60, 64), WIDER (Workgroup for Intervention Development and Evaluation Research) (1 study) (64), StaRI (Standards for reporting implementation studies) (1 study) (57). Among grey literature documents, only one (86) recommends the TIDieR checklist to help design and refine the intervention, as well as to describe it for dissemination and replication.

## 4 Discussion

This scoping review analysed 59 documents, comprising 49 scientific articles and 10 grey literature sources, to examine the evolution of audit and feedback (A&F) intervention design processes over fifteen years. The study focused on the theoretical frameworks chosen to guide the design process, and the staged structure described for that process.

We highlight several findings. First, the included scientific studies in the review utilised a wide range of theoretical frameworks, applying up to twenty-two different models, either individually or in combination.

Among the grey literature documents, six of them were based on specific design frameworks, encompassing five distinct models overall. Two further documents did not specify a theoretical framework (88, 89), and two left the choice to the design team (3, 86). This indicates good availability of documents to guide the design offering a high degree of methodological flexibility.

In terms of the number of stages identified in behaviour change interventions design, a second finding was the significant diversity in the literature, which ranged from two to ten, with a five-stage approach being the most common. Consistent with Colquhoun's review (8), which identified a maximum of seven stages and five core tasks, this review highlights ongoing heterogeneity in design processes. A key trend in recent years is the increased emphasis on defining actions to ensure the long-term sustainability of implementation outcomes. Our review of the grey literature identified variation in the number of stages across the different documents. In two of the documents (21, 89), these variations were justified because the document focused on specific stages of the design process rather than describing the entire process. In the remaining documents, the omitted stages were the development of a pilot study (3, 25, 84, 88), and the planning of actions to ensure the sustainability of long-term effects (88).

The ten selected grey literature documents consisted of nine methodological guidelines structured by design stage (21, 25, 84–88, 90), one focused specifically on the audit and feedback (A&F) approach (89). The tenth was a comprehensive review of the empirical basis for evidence-based healthcare that is both a valuable review and practical guide to theoretical frameworks, tools and techniques in implementation science (3). Regarding the choice of theoretical frameworks, its author recommends that design teams select a theoretical framework at the start of the design process and allows for a mixed-framework approach if appropriate in the context. While this accounts for the variety of options available, navigating these choices and selecting a theoretical framework poses a methodological challenge for design teams. To assist with this, tools such as SELECT-IT (Systematic Evaluation and Selection of Implementation Science Theories, Models and Frameworks) (92) have recently been developed.

A detailed analysis of the design process revealed that team formation is increasingly recognised as a critical success factor, with nearly all the documents and guidelines in our review identifying it as a formal initial stage. Although explicitly referenced in only 12 articles in our review – and absent from Colquhoun's 2017 review (8) – this shift highlights the growing need for a participatory approach and active stakeholder representation throughout the design life cycle. Grey literature documents advocate for embedding patients, carers, targeted professionals, and expert methodological advisers into the team from the outset. They also recommend including other stakeholders, such as implementation facilitators, in the team.

After initial team formation, our review confirms that defining objectives, identifying barriers and facilitators, and selecting intervention components are critical stages in the design process. These stages are cited in the scientific studies included in this review and are detailed across all the guidelines and documents identified. As noted in the 2017 review (8), executing these stages is standard in systematic design. While most guidelines make general recommendations for development, the BCW guideline focuses specifically on these stages, describing in detail the actions to be taken, along with examples and support tools for each stage. This makes it an invaluable practical manual for the design process. Other documents, such as those from the RNAO and JBI, also provide excellent tools to support specific activities linked to these stages, which could be extremely useful to intervention design teams starting out on the process. These include pre-defined questionnaires to help identify barriers and facilitators, and summaries of the effectiveness of potential intervention components.

Identifying barriers and facilitators is itself a distinct research process that requires specialist qualitative research expertise. Design teams therefore need to include professionals with this skill set, which can create challenges related to team training and resource availability.

Some grey literature documents (3, 25, 84–86) expand the typical intervention development process by adding a stage focused on searching for evidence to justify the need for the intervention and, where necessary, tailoring it to the local context. Although this stage was not always consistently reported in Colquhoun's 2017 review, our review identified twenty-one studies in the scientific literature that incorporated evidence searching at various points in the design process (41, 42, 50, 53, 57–59, 63, 64, 66, 70, 72, 74, 75, 77, 78, 80, 83). Three of these studies also reported adapting the evidence to the local context (37, 65, 79).

Monitoring and evaluation activities were addressed in six grey literature documents, but only slightly more than half of the scientific studies included in our review reported undertaking this stage. Colquhoun et al.'s (8) review found that this critical stage was often overlooked. Given that monitoring and evaluation are crucial for measuring implementation success, determining impact and informing redesign for scaling up, we argue that reinforcing this stage within the design process should be a priority area for improvement.

Further along in the design process, seven guidelines make recommendations for implementation planning. Several advise involving facilitators with expertise in co-creation and implementation science to support care teams during intervention development (93, 94). Some studies also integrate pilot or feasibility studies at this stage, recognising that interventions lacking feasibility directly hinder implementation (95). Pilot stages were rarely mentioned a decade ago and remain uncommon in study designs today, appearing in fewer than half of the published articles. While piloting can be costly and time-consuming, pilot studies are increasingly recognised as essential for reviewing, refining and transforming initial designs into viable initiatives, particularly when developing complex interventions. Dedicated guidelines now exist to structure this process (96). Not all grey literature sources systematically include pilot studies, although some documents recommend assessing the feasibility of individual intervention components to ensure robust implementation (84, 86, 90).

The final stage of the design process identified in the review is the development of strategies to ensure the long-term sustainability of the intervention. This reflects concerns that the immediate effects of interventions – the changes in clinical practice and resulting health benefits – often fade over time. Without planning for sustainability early in the design phase, rather than retrospectively, activities may cease and the capabilities fostered by the intervention may be lost (85, 97–100). Ultimately, failure to sustain initiatives not only wastes human and economic resources but also undermines confidence and enthusiasm for future improvement initiatives among professionals and other stakeholders, including patients. This stage is described in only eleven of the studies included in our review, although it is already addressed in most of the grey literature. Sustainability is closely linked to the understanding that implementing interventions in clinical practice is an iterative process (101), sometimes characterised as “never-ending”, in which results obtained serve as a reference for making proposals for continuous improvement (102). While the traditional view treats sustainability as a stage separate from implementation, more recent grey literature suggests it should be viewed as an ongoing component of both design and implementation rather than a final step. Benzies et al. (103) define sustainability as a continuous, dynamic stage within implementation, and emphasise that identifying strategic inflection points can accelerate the sustainability of an intervention.

As early as 2012, researchers recognised that a systematic, sequential design is critical to improving intervention effectiveness (5, 7). From a continuous quality improvement perspective, such structure is essential to ensure that interventions are designed, implemented, evaluated and refined in a consistent and reproducible manner. The variability in intervention design processes observed across the scientific literature likely reflects the use of different methodological guidelines and inconsistent adherence to their recommendations. This variability represents a missed opportunity to strengthen implementation and promote the consistent application of established design frameworks. Accordingly, applying a systematic design process to clinical practice interventions should be considered a core component of quality improvement rather than a methodological option. In resource-constrained settings, the use of established frameworks can enhance efficiency and increase the likelihood of developing effective and sustainable interventions. However, because teams operate under real-world clinical, organisational, and policy constraints, achieving an ideal design process is often not feasible. It is therefore essential to identify which design stages are indispensable and which can be adapted or streamlined according to context.

Furthermore, within this quality improvement perspective, transparency is a key enabling element, as it allows successful interventions to be disseminated and scaled up (104). A notable finding of this review was that only 8 of the 49 studies published since 2014 used established reporting guidelines, and only one grey literature document recommended their use. Intervention design teams have been encouraged to report their development processes in a detailed and systematic way to facilitate replication and adaptation to new contexts, making transparent reporting a fundamental requirement for effective quality improvement. Despite the availability of tools such as TIDieR and GUIDED, their uptake remains limited.

The grey literature included in our review exhibited several notable omissions. The first is the near absence of update protocols; the only exception is the BCW guide, which directs readers to an external website for development updates (https://www.humanbehaviourchange.org/) (105). As a result, it remains unclear how these documents will incorporate emerging evidence on the design process, or on the effectiveness of audit and feedback interventions, for which recent evidence is available in a recent Cochrane review (9). A second gap in the documents is the lack of attention to the substantial time investment required for full intervention development, an issue that is also systematically underreported in the scientific literature. Although development timelines will vary depending on available resources, including real-world design examples in guidelines would better support users and adopting organisations. Rapid design proposals using online Delphi techniques have recently been developed to address these time constraints (106). The third omission is the complete absence of advice or guidance on how to evaluate the cost-effectiveness of planned interventions; this essential consideration could be integrated into monitoring and evaluation or treated as a separate stage. Fourthly, although intervention adaptation is frequently necessary, SSB-Univ (86) was the only guideline in our review that explicitly addressed the possibility of adapting existing interventions. In practice, structured frameworks for adapting interventions to new contexts are already available, such as the ADAPT guidance (107). Complementary guidelines for the good reporting of intervention adaptations have also been developed, including the FRAME (Framework for Reporting Adaptations and Modifications to Evidence-Based Interventions) guidelines (108, 109). Fifthly, the grey literature reviewed lacks explicit guidance on whether implementation strategies should differ when introducing new practices versus de-implementing existing ones. Emerging evidence suggests that incorporating “unlearning components” (110, 111) is essential for effective de-implementation, arguing that implementation without unlearning results in accumulation rather than meaningful progress. Because unlearning is a complex process involving multiple factors –knowledge, workflows, record systems, standards, and the underlying evidence base, as well as the active reinforcement of new behaviours that replace old ones– identifying the core intervention components that facilitate disengagement is critical.

Finally, given that the grey literature documents analysed are intended to function as design guides, it would be helpful, particularly for groups just beginning this work, if they included a section with advice or recommendations for the gradual adoption of the systematic design methodology within an intervention. Such a section could outline key entry points and describe how to incorporate other stages of the methodology into future designs. Emphasising the possibility of gradual adoption would also convey that the approach perception that this approach is feasible.

### 4.1 Study strengths and limitations

This study is strengthened by its adherence to PRISMA guidelines and the use of a pre-registered protocol. To ensure rigour and minimise bias, research team members agreed on classification criteria, study selection and data extraction through consensus, with a third reviewer resolving any discrepancies. Furthermore, expanding the search to grey literature enabled us to identify and include detailed guidelines and recommendations for intervention design from recognised organisations and research groups with expertise and experience in implementation science.

Several limitations should be noted. First, as with any literature synthesis, our findings rely solely on published data and may not have captured underreported aspects of A&F intervention design. Second, language restrictions (English and Spanish) may have excluded relevant studies, potentially limiting the comprehensiveness of the review.

In addition, some grey literature documents may have been published as peer-reviewed articles rather than grey literature and therefore fell outside our search strategy or may have been published on websites without public access. Furthermore, because the aim was to describe the structure of the design process, the results are synthesised narratively, rather than statistically. Finally, this review focuses on intervention design processes within implementation science or quality improvement and does not include alternative approaches like complexity science or the social sciences (104).

## 5 Conclusions

Our scoping review of scientific and grey literature published over the past fifteen years shows persistent variability in the design interventions aimed at modifying clinical practice. This variability is reflected both in the theoretical models used to guide intervention development and in the number of stages involved, with multiple models identified and up to eleven distinct design stages reported across the literature. While the systematic design of interventions is essential for ensuring their effectiveness, this analysis of reported design processes in the scientific literature reveals substantial room for improvement in methodological rigour and in the transparency with which available reporting tools are used. The combination of methodological gaps and lack of transparency hinders replicability.

Although grey literature guidelines, manuals, and summaries of empirical evidence offer valuable frameworks for groups seeking to develop systematic interventions, they also highlight that this process is complex and resource-intensive. Given the need for technical leadership from trained professionals, as well as considerable organisational resources—including time—such initiatives require strong institutional commitment, particularly at the meso level, to ensure feasibility and sustainability. From an organisational perspective, maximising intervention effectiveness and avoiding inefficiency or duplication of effort requires investment in specific design and implementation capabilities, as well as in stable support structures and networks that enable collaborative and reusable design processes. Without such strategic investment, intervention development is likely to remain unsystematic, ineffective and unsustainable.

Finally, the scoping review process identified several areas for future research. These include identifying barriers and facilitators regarding the adoption of systematic intervention design by healthcare organisations, as well as initiatives to support the professionals responsible for such implementation. Another area for future research is evaluating the impact of using a systematic design process on both intervention effectiveness and cost-effectiveness. Likewise, further work is needed to develop specific methodological guidelines for de-implementation.

Similarly, regarding methodology, it would be highly beneficial to have guidance documents on intervention design that incorporate diverse, multidisciplinary perspectives — such as those from complexity theory or the social sciences — in order to strengthen intervention design and best practices.

## Data Availability

The raw data supporting the conclusions of this article will be made available by the authors, without undue reservation.
