# Supplementary material for: Methods used to design audit and feedback interventions to change clinical practice: a scoping review
Source: Front Health Serv. 2026 Sep 15;6:1907196. doi: 10.3389/frhs.2026.1907196 (PMC13619827; doi:10.3389/frhs.2026.1907196)
Supplement: Supplementary file 2 [file Supplementaryfile2.docx]

# Supplementary file 2: Search strategies

**Supplementary file 2**. Databases search strategies

The search was carried out in three search cycles: from April 2015 to July 2023, from August 2023 to December 2023 and from January 2024 to October 2025. We show last search strategy on each database.

**Medline**

**Date of search: 26^th^ October 2025**

**Database: Ovid MEDLINE(R) and Epub Ahead of Print, In-Process, In-Data-Review & Other Non-Indexed Citations, Daily and Versions <1946 to October 24, 2025>**
**Search Strategy:**
**1**  Practice Patterns, Physicians'/
**2**  exp Evidence-Based Practice/
**3**  (chang* adj3 (behavio?r or practice)).tw.
**4**  (professional adj3 (behavio?r or practice)).tw.
**5**  "evidence based".tw.
**6**  implementation.tw.
**7**  or/1-6
**8**  (develop* or design*).ti.
**9**  (theory or theories or theoretical).ti.
**10**  (mechanism? or approach* or method*).ti.
**11**  (framework or constructs or process*).ti.
**12**  (tools or technique* or content or strateg*).ti.
**13**  or/8-12
**14**  intervention?.ti.
**15**  (intervention? adj3 (chang* or design* or develop* or behavior?r)).tw.
**16**  intervention study/
**17**  or/14-16
**18**  7 and 13 and 17
**19**  exp Clinical Audit/
**20**  Feedback/
**21**  (audit adj2 feedback).af. or A&F.ti,ab.
**22**  atrial fibrillation.ti,ab.
**23**  21 not 22
**24**  (implement* adj3 (intervent* or evidence* or reseach* or practic*or scienc*)).ti,ab.
**25**  Translational Medical Research/
**26**  19 or 20 or 23 or 24 or 25
**27**  18 and 26)
**28**  (A&F adj3 intervention*).ti,ab.
**29**  28 not 22)
**30**  27 or 29
**31**  limit 30 to (clinical conference or congress)
**32**  30 not 31
**33**  limit 32 to yr="2024 -Current"
**34**  "audit and feedback".af.
**35**  33 and 34 (39)

**EMBASE:** Plataforma Embase.com

**Date of search: 26 octubre 2025**

#1. 'clinical practice'/de OR 'evidence based practice'/exp

#2. (chang* NEAR/3 (behavio?r OR practice)):ti,ab,kw

#3. (professional NEAR/3 (behavio?r OR practice)):ti,ab,kw

#4. 'evidence based':ti,ab,kw

#5. implementation:ti,ab,kw

#6. #1 OR #2 OR #3 OR #4 OR #5

#7. develop*:ti OR design*:ti

#8. theory:ti OR theories:ti OR theoretical:ti

#9. mechanism?:ti OR approach*:ti OR method*:ti

#10. framework:ti OR constructs:ti OR process*:ti

#11. tools:ti OR technique*:ti OR content:ti OR strateg*:ti

#12. #7 OR #8 OR #9 OR #10 OR #11

#13. intervention?:ti

#14. (intervention? NEAR/3 (chang* OR design* OR develop* OR behavio?r)):ti,ab,kw

#15. 'intervention study'/de

#16. #13 OR #14 OR #15

#17. #6 AND #12 AND #16

#18. 'clinical audit'/exp

#19. 'feedback system'/de OR 'negative feedback'/de OR 'positive feedback'/de OR 'psychological feedback'/exp

#20. (audit NEAR/2 feedback):ti,ab,kw

#21. 'a&f':ti,ab

#22. 'atrial fibrillation':ti,ab

#23. #21 NOT #22

#24. (implement* NEAR/3 (intervent* OR evidence* OR research* OR practic* OR scienc*)):ti,ab

#25. 'translational research'/exp

#26. #18 OR #19 OR #20 OR #23 OR #24 OR #25

#27. #17 AND #26

#28. ('a&f' NEAR/3 intervention*):ti,ab

#29. #28 NOT #22

#30. #27 OR #29

#31	#30 AND [2024-2025]/py

#32	#30 AND [30-01-2024]/sd NOT [27-10-2025]/sd

#33	#31 OR #32

#34	#33 AND [embase]/lim NOT ([embase]/lim AND [medline]/lim)

#35	#34 NOT 'conference abstract'/it

**APA Psycinfo.**. Platform: Ebsco Research Databases

| S1 | audit and feedback | Proximity  Filters: 01/01/2024 - 10/26/2025 |
| --- | --- | --- |
